# Genomic Convergence toward Diploidy in Saccharomyces cerevisiae

**DOI:** 10.1371/journal.pgen.0020145

**Published:** 2006-09-22

**Authors:** Aleeza C Gerstein, Hye-Jung E Chun, Alex Grant, Sarah P Otto

**Affiliations:** Department of Zoology, University of British Columbia, Vancouver, British Columbia, Canada; Washington University, United States of America

## Abstract

Genome size, a fundamental aspect of any organism, is subject to a variety of mutational and selection pressures. We investigated genome size evolution in haploid, diploid, and tetraploid initially isogenic lines of the yeast *Saccharomyces cerevisiae.* Over the course of ~1,800 generations of mitotic division, we observed convergence toward diploid DNA content in all replicate lines. This convergence was observed in both unstressful and stressful environments, although the rate of convergence was dependent on initial ploidy and evolutionary environment. Comparative genomic hybridization with microarrays revealed nearly euploid DNA content by the end of the experiment. As the vegetative life cycle of S. cerevisiae is predominantly diploid, this experiment provides evidence that genome size evolution is constrained, with selection favouring the genomic content typical of the yeast's evolutionary past.

## Synopsis

Genome size is a fundamental aspect of all species and has the potential to influence a number of individual characteristics such as cell size, generation time, ecological tolerances, and reproductive traits. Although genome sizes range widely among species, the forces shaping the evolution of genome size are only poorly known. Here we provide the results of an ~1,800 generation evolution experiment using lines of the budding yeast S. cerevisiae with either one, two, or four copies of their genome (haploid, diploid and tetraploid, respectively). We found, surprisingly, that all haploid and tetraploid lines converged toward diploidy, the historical state of *S. cerevisiae,* by the end of the experiment. Further experiments suggest that entire sets of chromosomes were lost as genome size changed from tetraploid to diploid. Our results suggest that genome size is constrained by selection acting against changes from the historical genome size.

## Introduction

Organisms vary tremendously in genome size [1,2] yet the key evolutionary forces acting to shape genome size in any particular organism remain unclear. Genome size is subject to small-scale changes (gene insertions or deletions) as well as large-scale ploidy differences (changes in the number of full chromosome sets). Genome size is known to influence a variety of phenotypes, including cell size [3], generation time [4], ecological tolerances [5], and reproductive traits [6]. Gene copy number is also thought to affect long-term rates of evolution, by altering the available number of mutations [7] and the efficacy of selection [6,8,9]. Using the budding yeast Saccharomyces cerevisiae as a model system, experimental evolution studies have confirmed the influence of genome size on long-term rates of evolution [7,10,11]. Genomic composition can, in turn, evolve over the course of such experiments. Recent experiments provide strong evidence that genomic changes, including insertions, deletions, and translocations, contribute to adaptation to novel environments in both Escherichia coli [12] and S. cerevisiae [13].

To investigate the evolutionary importance of genome size, we evolved initially isogenic haploid, diploid, and tetraploid S. cerevisiae for 1,766 asexual generations in batch culture. Five replicate lines of each ploidy level were grown in two experimental environments: an unstressful medium consisting of standard lab YPD (yeast extract peptone dextrose), and a salt-stressed medium consisting of YPD and 0.6 M NaCl. Large-scale (ploidy level) changes in genome size throughout the timescale of the experiment were identified using flow cytometry, while relative changes in gene copy number, including aneuploidies and indels, were identified using comparative genomic hybridization (CGH) of genomic DNA to microarrays at the final time point. Remarkably, we found convergent evolution among initially haploid and initially tetraploid lines toward diploidy, the predominant vegetative state of S. cerevisiae [14]. These results suggest that genome size is subject to evolutionary inertia, with selection opposing shifts in ploidy away from the historical level.

## Results/Discussion

**Figure 1:**
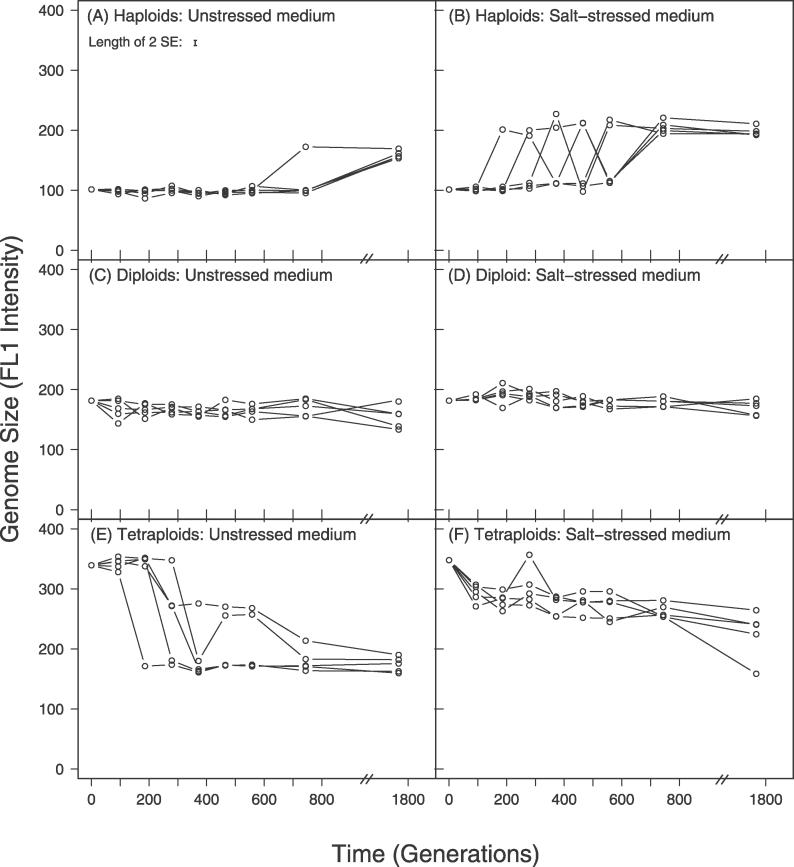
A Snapshot of Genome Size Change across 1,766 Generations of Batch Culture Evolution Each data point is the mean of three FACScan measurements on a single colony sampled from a population. Apparent fluctuations are largely a result of genome size polymorphisms, leading to sampling fluctuations depending on which colony was randomly chosen (see Figure S1). The average standard error (shown in panel A) reflects measurement error. FL1 represents a linear scale of dye fluorescence as measured by flow cytometry. The five lines on each graph represent the five replicate lines evolved independently.

Evolution with respect to genome size was surprisingly consistent: all strains converged toward or remained diploid (Figure 1), the predominant vegetative state of *S. cerevisiae.* Diploid individuals appeared and rose to high frequency through all ten replicate haploid populations (25 colonies were sampled from each line at generation 1,766; only one out of 250 colonies was still haploid), in both unstressed (Figure 1A) and salt-stressed (Figure 1B) media. Similarly, all ten initially tetraploid lines decreased in genome size (unstressed medium, Figure 1E; salt-stressed medium, Figure 1F). Cells of approximately diploid DNA content were found in all 5 × 25 colonies sampled at generation 1,766 from the five unstressed medium lines and from 25 colonies sampled from one of the salt-stressed lines (line qs), while cells of approximately triploid DNA content were observed in the 4 × 25 colonies sampled from the remaining four salt-stressed lines. Considerable polymorphism for genome size was apparent at earlier time points in this experiment for both initially haploid and initially tetraploid lines (Figures 1 and S1). Diploid lines showed no large-scale changes, though smaller-scale fluctuations in genome size occurred throughout the time series in both unstressed (Figure 1C) and salt-stressed (Figure 1D) media. The pattern of convergence towards diploidy was confirmed in a second independent experiment (Figure S2).

**Figure 2:**
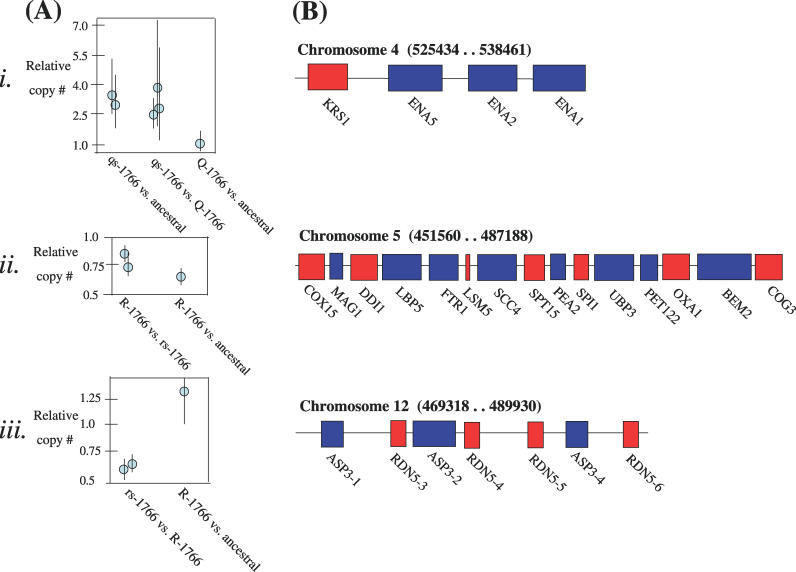
The Three Indels Identified by CGH Analysis of Ancestral (Generation 0) and Evolved (Generation 1,766) Tetraploid Lines (i) An insertion of a ~13-kilobase fragment on Chromosome 4 in tetraploid salt line qs; (ii) a potential ~36-kilobase deletion of Chromosome 5 in tetraploid line R; (iii) a potential 20-kilobase deletion of Chromosome 12 in tetraploid salt line rs. (A) Results of CGH, where each dot represents the mean (bars: 95% CI) relative copy number of all genes in the indel from a single array. (B) Genes of known function (http://www.yeastgenome.org 2 October 2005) affected by the indel (basepair range of genes involved are given in brackets). Each box is one ORF, where red indicates transcription on the Watson strand, and blue for genes transcribed on the Crick strand.

To find out whether chromosomes were present in euploid or aneuploid ratios in the evolved tetraploid lines, CGH was performed. Chromosomal content of the tetraploid cells was generally close to euploid, regardless of the samples compared. A euploid index was calculated from each array comparison where 1 indicates euploidy, while each chromosome deviating substantially (>10%) from the expected ratio reduces the index by 1/16. The euploid index was 0.891 between pairs of samples compared from early in the experiment (within the first 200 generations; Figure S3), 0.896 for samples compared from the end of the experiment (generation 1,766) to ancestral samples (generation 0, Figure S4), and 0.927 for two samples from the end of the experiment (Figure S5; estimates are likely to be biased downward; see Protocol S1). Although minor or partial aneuploidy may have been present in the initial lines, we did not detect any missing regions in the microarray analysis, indicating that no large-scale aneuploidy was fixed in the initial lines. Thus, while some aneuploidy was present at the end of our experiment, it typically involved few chromosomes (often Chromosome 9) and was not consistent with random chromosome loss. Interestingly, CGH also detected at least three indels (Figure 2A, Figure S6). Of particular interest was a duplication within one of the tetraploid lines reared in salt-stressed (Figure 2Ai, line qs), encompassing several genes on Chromosome 4 involved in sodium efflux (the P-type ATPases, *ENA1* (YDR040C), *ENA2* (YDR039C), and *ENA5* (YDR038C)). Deletions within Chromosome 5 (Figure 2Aii) and Chromosome 12 (Figure 2Aiii) were also identified (Protocol S1).

The driving force behind the genomic size decrease in tetraploid lines could have been selection or mutational bias (due to deletions outnumbering insertions). To distinguish between these possibilities, we conducted a bottleneck experiment in unstressed medium starting from the same ancestral tetraploid culture. By reducing the population size every 48 h to a single cell by picking and streaking single random colonies, we limited the variability necessary for selection to act. Consequently, mutational biases and drift became the dominant evolutionary forces. Results were significantly different from the original experiment. After 572 generations (26 bottlenecks), diploids were present in only two of ten bottlenecked lines compared with five out of five lines evolved at large population sizes over this same time period in the original experiment (Fisher's exact test, *p* = 0.007; Figure S7). The average genome size observed in the primary experiment (181.48 FL1 intensity ± 7.87) was also significantly lower (t_11_ = 4.697, *p* = 0.0003) than the average genome size from the bottlenecked populations (286.06 ± 20.83). Therefore, selection on genome size is required to account for the rapid convergence toward diploidy observed in Figure 1.

**Figure 3:**
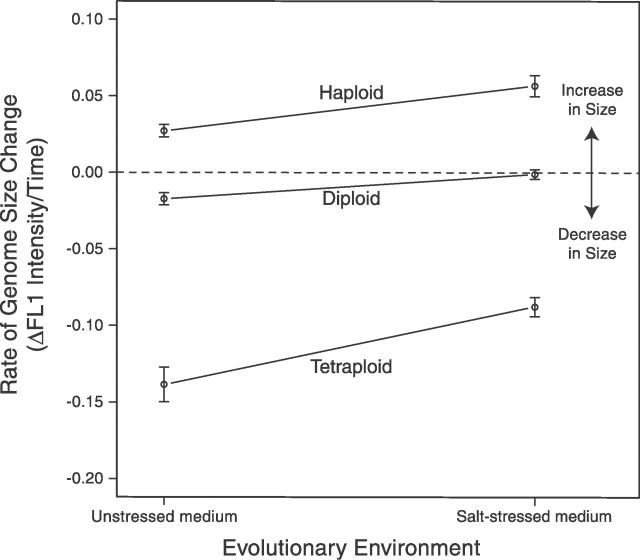
Rate of Genomic Size Change by Ploidy and Environment Rate of change was calculated by fitting linear regression lines through timeseries data (Figure 1) for each individually evolved line. Each data point thus represents the mean ± SE of five slope measures. This figure shows that haploids increased in genome size faster in salt, while tetraploids decreased in genome size more slowly in salt.

Ploidy level and environment showed a significant interaction (F_2,24_ = 3.595, *p* = 0.0431) on the pattern of genome size evolution (Figure 3). Initially, haploid lines increased in genome size faster in salt-stressed medium (t_8_ = 3.729, *p* = 0.0058), while initially tetraploid lines decreased in genome size slower in salt-stressed medium (t_8_ = −3.948, *p* = 0.0042). Although the diploid strains showed only small-scale decreases in genome size, the rate of loss was also significantly more rapid in unstressed medium (t_8_= −3.2715, *p* = 0.011).

Because the lines used in this study lack a pheromone receptor required for mating and carry a mutation-preventing sporulation, syngamy and meiosis should not have occurred. To ensure that mate switching did not occur in the haploid lines (which might have allowed for sexual reproduction), PCR of the mating type (MAT) locus was performed. We found that only the MATa allele was present at this locus in all haploid-evolved lines (generation 1,766), arguing against mate switching. We also performed a sporulation assay using known protocols [15] on all lines at the end time point (1,766 generations). No spores were found in any experimental lines, though sporulation was observed in a positive control using a yeast strain known to sporulate [16]. Thus, it is unlikely that meiosis occurred during the course of the experiment. One plausible mechanism for mitotically occurring genome size change is that haploid individuals in both unstressed and salt-stressed media underwent endomitosis (chromosome replication not followed by division), creating diploid offspring with two copies of each haploid parental gene. The process by which tetraploids lost DNA remains unclear, but appears to follow roughly euploid shifts in chromosome content. The time frame over which we observed euploid shifts in DNA content from tetraploid to diploid was short (100–200 generations, Figure 1). For 32 chromosomes to be lost in rapid succession within such a short time frame, there would had to have been at least a 550% fitness gain each time a chromosome was lost (Protocol S1). As the initial growth rate of the tetraploid lines was only marginally reduced (by 5%–10% relative to haploids and diploids), it is highly unlikely that the transition to diploidy involved the independent appearance and selective spread of cells that lost one chromosome at a time. This argues for a concerted mutational process involving the loss of multiple chromosomes, generating mutant cells that are approximately euploid and that are selectively favoured (as demonstrated by the bottleneck experiment; Figure S5). Such rapid and concerted loss of multiple chromosomes has been observed in Candida albicans [17], a historically diploid yeast species closely related to S. cerevisiae.

Typically, models of ploidy evolution predict that either larger genomes or smaller genomes are favoured, depending on the environment, population size, and reproductive system of the organism [6,18]; intermediate ploidy levels are not generally expected in the absence of constraints. Yet in our study we find strong, repeated evidence for selection on diploidy, the intermediate ploidy level.

As S. cerevisiae is historically diploid, we conjecture that selection has acted over evolutionary time to optimize organismal function with two full sets of chromosomes. After a period of time at a particular ploidy level, an organism might become well adapted to the attendant cell size and gene expression patterns, reducing the fitness of ploidy mutants. For example, even isogenic S. cerevisiae strains of different ploidy levels exhibit altered gene expression patterns [19], which might select against lines with novel ploidy levels. We hypothesize that diploids potentially have a competitive advantage over haploids. Although no significant competitive differences were detected by a different study using the same strain [20], the power of this study was such that a small difference in competitive ability (i.e., 10%) could not have been detected. Consistent with our hypothesis, a study comparing haploid and diploid individuals of historically haploid *(Schizosaccharomyces pombe)* and diploid *(S. cerevisiae)* yeast found that evolutionary history, rather than environmental conditions, predicted individual competitive performance and growth rates (V. Perrot, personal communication). Whether convergence toward haploidy would be observed in historically haploid yeast remains to be seen and would allow us to distinguish between evolutionary inertia or historical constraint versus a generalized advantage of diploidy.

The rate of convergence toward diploidy was highest for haploids in salt-stressed medium and tetraploids in unstressed medium. Coupled with our finding that initially tetraploid lines in the salt-stressed medium have a significantly higher genome size at generation 1,766 relative to initially tetraploid lines grown in unstressed medium (t_8_ = 2.75, *p* = 0.025), we conclude that a large genome size was slightly more favourable in the salt-stressed medium. This result suggests that the adaptive benefits of higher or lower genome size are affected by the ecological environment. Thus, while ploidy itself might be constrained by historical factors, the rate of adaptation with respect to genome size change is likely influenced by the environment.

Here we have shown that an intermediate ploidy is selectively favoured in two different environments. Our results suggest that evolutionary inertia might act to constrain genome size evolution, preventing shifts away from the ploidy level to which an organism has historically adapted.

## Materials and Methods

### Generation of lines.

Haploid, diploid, and tetraploid lines of S. cerevisiae were initiated from culture frozen down by B. Mable [18] (strains BM1N, BM2N, and BM4N descended from haploid strain SM2185 kindly provided by A. Adams and S. Brower). These three lines were isogenic with haplotype *MATa-a1 ste6*Δ*8–694 ura3 leu2 his4 trp1 can1*. A deletion in the pheromone receptor locus *(ste6*Δ*8–694)* and a mutation in the MAT locus (*MATa,* mutation *a1*) should prevent mating [21] and sporulation [22], respectively, and eliminate any potential pleiotropic effects of the MAT locus on relative fitness [23].

### Batch culture evolution.

Ancestral ploidy lines were initiated by streaking frozen stock (BM1N, BM2N, and BM4N) onto YPD plates, picking off a single colony after 48 h, and culturing for 24 h in the appropriate (unstressed or salt-stressed) liquid medium. Unstressed medium was YPD (Difco, Sparks, Maryland, United States). The salt-stressed medium was unstressed medium plus 0.6 M NaCl, which reduced initial growth rates by 35%. Culture was then frozen down at −80 °C in 15% dimethyl sulfoxide (DMSO; Sigma, St. Louis, Missouri, United States) as the six time zero lines (three ploidy levels × two environments). 5 × 100 ul from each of these six initial tubes was then pipetted into 10 ml of appropriate medium and used to initiate five replicate lines. 266 daily (24 h ± 1 h) 1:100 transfers (100 μl culture into 10 ml medium) were conducted sequentially. As each transfer allowed ~6.64 mitotic divisions (2^6.64^ = 101) before the population returned to stationary phase, a total of ~1,766 (= 266 × 6.64) cell generations occurred per line regardless of environment. Cultures were continually shaken at 200 rpm and maintained at 30 °C overnight. Subsequent freezing was conducted every 2 wk (93 generations).

### Genome size determination.

Flow cytometry (FACScans) was used to determine relative ploidy of all lines at nine different time points: 0, 93, 186, 279, 372, 465, 558, 744, and 1,766 cell generations. The FACScan protocol [24] was modified as described by the Fred Hutchinson Cancer Research Center (http://www.fhcrc.org/science/labs/gottschling/yeast/facs.html). Cells from frozen culture were streaked to single colonies on YPD plates. A single colony was picked off and grown in YPD for 24 h. The only deviation in protocol was that the last pellet was resuspended in 980 μl of sodium citrate and 20 μl of 0.05 mM SYTOX Green dye. Cultures were kept at room temperature for a minimum of 3 h (but up to 24 h) to ensure dye uptake and then stored at 4 °C overnight.

30,000 cells from each culture were analyzed on a FACSCalibur (Becton-Dickinson Immunocytometry Systems, Palo Alto, California, United States). The FL1 detector was used for the acquisition of SYTOX Green fluorescence, where dye is taken up by the cells in a manner stoichiometric to the amount of DNA in the nucleus. The FlowJo (Tree Star, Ashland, Oregon, United States) cell cycle analysis function using the Watson pragmatic option was used to fit Gaussian curves to our data to determine the FL1 intensity corresponding to the G1 mean, which indicates the average unreplicated DNA content of each population of cells [25].

FACScans over all time points for the five replicate lines of each treatment (45 tubes) were performed on the same day. The entire protocol was replicated on three different days using cells from the same colonies; any variation reflects machine/treatment variation and not genetic variation within the cultures. A significant day effect was found, and the data were corrected by adjusting the replicate data collected on different days to have the same mean. The corrected data were used for subsequent analyses.

### Microarrays.

On a subset of the tetraploid lines, we used CGH of genomic DNA of microarrays to determine whether the evolved lines were euploid or aneuploid [26,27]. For each CGH, a colony was isolated from frozen stock and grown to stationary phase in liquid YPD. Genomic DNA was extracted from 8 ml of stationary phase culture using a standard yeast mini-prep DNA isolation procedure [28]. Genomic DNA (5 μg) was sonicated (3 × 10 s at 45% of 20 kHz) to obtain DNA fragments of roughly 100 basepairs to 10 kilobases and purified with a QIAquick PCR Purification Kit (Qiagen, Valencia, California, United States). The two DNA samples to be compared were labeled with Cy3 or Cy5 using the Mirus Label IT Nucleic Acid Labeling Kit (Mirus, Madison, Wisconsin, United States), according to the manufacturer's protocol. We then co-hybridized the labeled genomic DNA to S. cerevisiae microarrays obtained from the University Health Network Microarray Centre (Toronto, Ontario, Canada). The hybridized slides were washed and scanned using ScanArray Express (PerkinElmer, Wellesley, California, United States) set to the yeast protocol. QuantArray (PerkinElmer) was used to quantify the relative fluorescence of Cy3 and Cy5 between the two samples of interest. Finally, GeneSpring (Agilent, Palo Alto, California, United States) was used to order the data according to chromosomal location.

### CGH analysis.

All fluorescence ratios were log-transformed prior to analysis and back-transformed for presentation. The average fluorescence ratio was first calculated for each chromosome to assess the degree of aneuploidy. A 99.8% confidence interval for the chromosomal average ratio was obtained by bootstrapping. Bootstrapping involved randomly sampling from the gene ratios observed within a particular chromosome with replacement, yielding a bootstrap dataset with the same number of data points as the original chromosome; 1,000 bootstrap datasets were obtained per chromosome. A 99.8% confidence interval was chosen to correct for multiple comparisons across the 16 chromosomes of S. cerevisiae (giving an overall alpha value per genome of α = 0.03).

As the same concentration of DNA was hybridized to each microarray, a CGH analysis cannot assess relative differences in ploidy level between lines. Aneuploidy of a chromosome can be detected, however, as a departure from a fluorescence ratio of one in a CGH comparison of two otherwise euploid genomes. A decrease in copy number of a particular chromosome is expected to lead to a 0.5 ratio (in diploids), 0.67 (in triploids), or 0.75 ratio (in tetraploids) relative to the rest of the chromosomes. Conversely, an increase in copy number of a particular chromosome is expected to lead to a 1.5 ratio (in diploids), 1.33 ratio (in triploids), or 1.25 ratio (in tetraploids) relative to the rest of the chromosomes.

### Bottleneck experiment.

Ten replicate tetraploid populations were streaked onto YPD plates. Every 2 d (~22 generations) of growth, a single random colony was picked and streaked onto a new plate. Culture was frozen every 2 wk (~154 generations). This procedure of repeated bottlenecks ensured that each line had a low effective population size [29] (N_e_ = 22).

### Rate of genome size evolution.

A regression line was fit through the genome size data as a function of time. The y-intercept was constrained as the genome size at time zero and was thus the same for all ten (replicates × environment) lines of each ploidy. The mean slope was calculated for each of the six populations (ploidy × environment) as the mean of the slopes of the five replicate lines. A two-way ANOVA was performed to test for an interaction between ploidy and environments. *t*-tests were then performed to determine differences in the rate of genome size evolution between environments for each ploidy level. The assumption of normality was met in all cases (*p* > 0.05). All analyses were performed using JMP [30].

### PCR.

Yeast genomic DNA was isolated. PCR was run twice for all evolved (generation 1,766) haploid lines and the ancestral haploid lines using forward primers specific to the MATa (5′–CTCCACTTCAAGTAAGAGTTTGGGT–3′) and MATalpha (5′–TTACTCACAGTTTGGCTCCGGTGT–3′) alleles and a common reverse primer (MAT 3′–R: 5′–GAACCGCATGGGCAGTTTACCTTT–3′). Amplification of DNA sequence was achieved by 30 cycles of DNA denaturation (96 °C for 1 min), primer annealing (55 °C, 1 min), and primer elongation (72 °C, 1 min) followed by a 5-min incubation at 72 °C after the final cycle. The haploid yeast strains YPH 499 (MATa) and YPH 500 (MATalpha) were used as controls to ensure the primers amplified the proper regions.

## Supporting Information

Figure S1 Temporal Polymorphism for Genome Size across 1,800 Generations of Batch Culture (725 KB PDF)

Figure S2 Genome Size Change across ~600 Generations of Batch Culture Evolution from a Replicate Experiment (214 KB PDF)

Figure S3 Average Fluorescence Ratio by Chromosome from CGH between Lineages Early in the Experiment (580 KB PDF)

Figure S4 Average Fluorescence Ratio by Chromosome from CGH between Lineages Late and Early in the Experiment (631 KB PDF)

Figure S5 Average Fluorescence Ratio by Chromosome from CGH between Lineages Late in the Experiment (624 KB PDF)

Figure S6 Sliding Window Analysis of the Arrays, using a Window Length of Ten Genes (4.7 MB PDF)

Figure S7 Genome Size Measured from Ten Populations (B1–B10) Evolved by Repeated Bottlenecking for 566 Generations and Five Populations (S1–S5) Evolved through Batch Culture Transfers for 558 Generations (542 KB PDF)

Protocol S1 Supplementary Material (46 KB DOC)

### Accession Numbers

The *Saccharomyces* Genome Database (SGD) (http://www.yeastgenome.org) accession numbers for Chromosomes 4, 5, and 12 are NC_001136.8, NC_001137, and NC_001144.4, respectively; the accession numbers for *ENA1, ENA2,* and *ENA5* are YDR040C, YDR039C, and YDR038C, respectively.

## Abbreviations

CGH: comparative genomic hybridization
MAT: mating type
YPD: yeast extract peptone dextrose

## References

[pgen-0020145-b001] Cavalier-Smith T (1978). Nuclear volume control by nucleoskeletal DNA, selection for cell volume and cell growth rate, and the solution of the DNA C-value paradox. J Cell Sci.

[pgen-0020145-b002] Gregory TR (2001). Coincidence, coevolution, or correlation? DNA content, cell size, and the C-value enigma. Biol Rev.

[pgen-0020145-b003] Weiss RL, Kukora JR, Adams J (1975). The relationship between enzyme activity, cell geometry, and fitness in Saccharomyces cerevisiae. Proc Natl Acad Sci U S A.

[pgen-0020145-b004] Petrov DA (2001). Evolution of genome size: New approaches to an old problem. Trends Genet.

[pgen-0020145-b005] Lewis WH (1980). Polyploidy: Biological relevance.

[pgen-0020145-b006] Otto SP, Whitton J (2000). Polyploid incidence and evolution. Annu Rev Genet.

[pgen-0020145-b007] Paquin C, Adams J (1983). Frequency of fixation of adaptive mutations is higher in evolving diploid than haploid yeast populations. Nature.

[pgen-0020145-b008] Perrot V, Richerd S, Valero M (1991). Transition from haploidy to diploidy. Nature.

[pgen-0020145-b009] Orr HA, Otto SP (1993). Does diploidy increase the rate of adaptation?. Genetics.

[pgen-0020145-b010] Zeyl C, Vanderford T, Carter M (2003). An evolutionary advantage of haploidy in large yeast populations. Science.

[pgen-0020145-b011] Anderson JB, Sirjusingh C, Ricker N (2004). Haploidy, diploidy and evolution of antifungal drug resistance in Saccharomyces cerevisiae. Genetics.

[pgen-0020145-b012] Riehle MM, Bennett AF, Long AD (2001). Genetic architecture of thermal adaptation in Escherichia coli. Proc Natl Acad Sci U S A.

[pgen-0020145-b013] Dunham MJ, Badrane H, Ferea T, Adams J, Brown PO (2002). Characteristic genome rearrangements in experimental evolution of Saccharomyces cerevisiae. Proc Natl Acad Sci U S A.

[pgen-0020145-b014] Nasmyth K, Shore D (1987). Transcriptional regulation in the yeast life cycle. Science.

[pgen-0020145-b015] Kassir Y, Simchen G (1991). Monitoring meiosis and sporulation in Saccharomyces cerevisiae. Methods in Enzym.

[pgen-0020145-b016] Zeyl C, deVisser JA (2001). Estimates of the rate and distribution of fitness effects of spontaneous mutation in Saccharomyces cerevisiae. Genetics.

[pgen-0020145-b017] Bennett RJ, Johnson AD (2003). Completion of a parasexual cycle in Candida albicans by induced chromosome loss in tetraploid strains. EMBO J.

[pgen-0020145-b018] Mable BK, Otto SP (2001). Masking and purging mutations following EMS treatment in haploid, diploid and tetraploid yeast (*Saccharomyces cerevisiae)*. Genet Res.

[pgen-0020145-b019] Galitski T, Saldanha AJ, Styles CA, Lander ES, Fink GR (1999). Ploidy regulation of gene expression. Science.

[pgen-0020145-b020] Mable BK (2001). Ploidy evolution in the yeast Saccharomyces cerevisiae: A test of the nutrient limitation hypothesis. J Evol Biol.

[pgen-0020145-b021] Kuchler K, Sterne RE, Thorner J (1989). *Saccharomyces cerevisiae STE6* gene product: A novel pathway for protein export in eukaryotic cells. EMBO.

[pgen-0020145-b022] Kassir Y, Hicks JB, Herskowitz I (1983). *SAD* Mutation of Saccharomyces cerevisiae is an extra **a** cassette. Mol Cell Biol.

[pgen-0020145-b023] Selk E, Wills C (1998). Mismatch repair and the accumulation of deleterious mutations influence the competitive advantage of *MAT* (mating type) heterozygosity in the yeast Saccharomyces cerevisiae. Genet Res Camb.

[pgen-0020145-b024] Nash R, Tokiwa G, Anand S, Erickson K, Futcher AB (1998). The WHI1+ gene of Saccharomyces cerevisiae tethers cell division to cell size and is a cyclin homolog. EMBO J.

[pgen-0020145-b025] Watson JV, Chambers SH, Smith PJ (1987). A pragmatic approach to the analysis of DNA histograms with a definable G1 peak. Cytometry.

[pgen-0020145-b026] Pollack JR, Perou CM, Alizadeh AA, Elsen MB, Pergamenschikov A (1999). Genome-wide analysis of DNA copy-number changes using cDNA microarrays. Nat Genet.

[pgen-0020145-b027] Hughes TR, Roberts CJ, Dai H, Jones AR, Meyer MR (2000). Widespread aneuploidy revealed by DNA microarray expression profiling. Nat Genet.

[pgen-0020145-b028] Davis RW, Thomas M, Cameron J, St John TP, Scherer S (1980). Rapid DNA isolation for enzymatic and hybridization analysis. Methods Enzymol.

[pgen-0020145-b029] Gerrish PJ, Wahl LM (2001). The probability that beneficial mutations are lost in populations with periodic bottlenecks. Evolution.

[pgen-0020145-b030] SAS Institute (2005). JMP 5.1.

