# Supplementary material for: Genomic Convergence toward Diploidy in Saccharomyces cerevisiae
Source: PLoS Genet. 2006 Sep 22;2(9):e145. doi: 10.1371/journal.pgen.0020145 (PMC1570378; doi:10.1371/journal.pgen.0020145)
Supplement: Supplementary file 1 [file pgen.0020145.sg001.pdf]

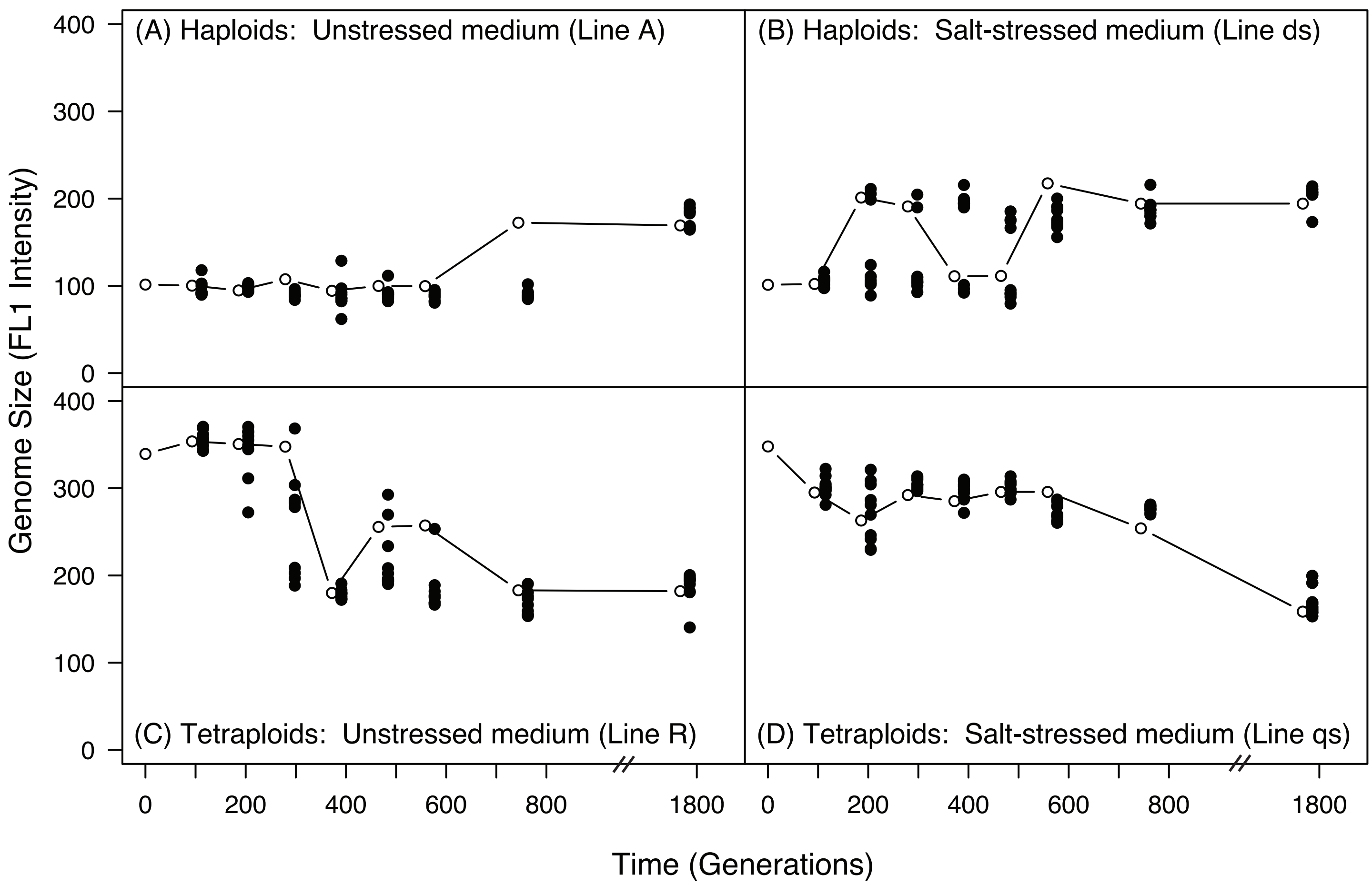

(A) Haploids: Unstressed medium (Line A)
(B) Haploids: Salt-stressed medium (Line ds)
(C) Tetraploids: Unstressed medium (Line R)
(D) Tetraploids: Salt-stressed medium (Line qs)
Genome Size (FL1 Intensity)
0
100
200
300
400
0
200
400
600
800
1800
Time (Generations)
