# Supplementary material for: Genomic Convergence toward Diploidy in Saccharomyces cerevisiae
Source: PLoS Genet. 2006 Sep 22;2(9):e145. doi: 10.1371/journal.pgen.0020145 (PMC1570378; doi:10.1371/journal.pgen.0020145)
Supplement: Supplementary file 2 [file pgen.0020145.sg002.pdf]

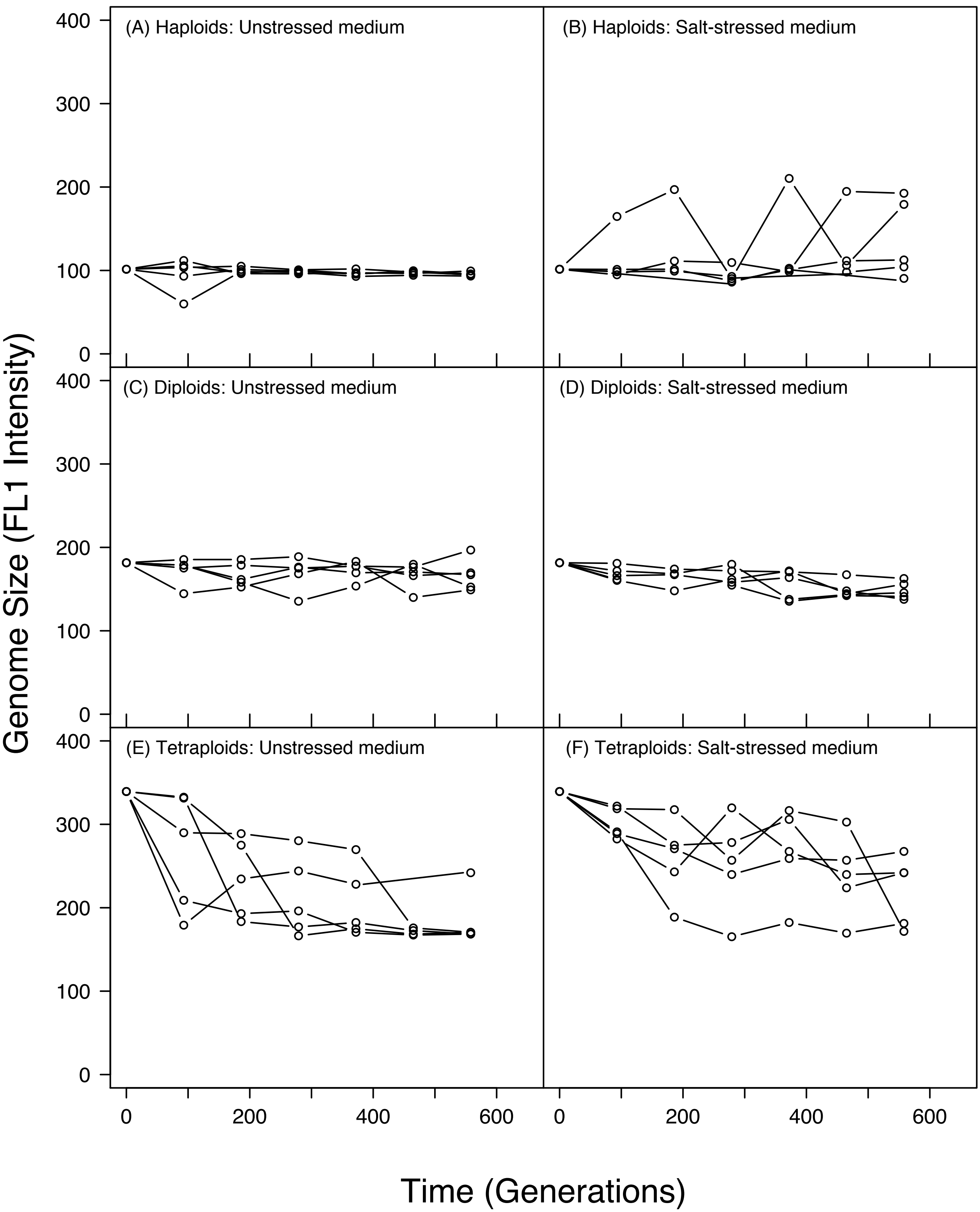

(A) Haploids: Unstressed medium
(B) Haploids: Salt-stressed medium
(C) Diploids: Unstressed medium
(D) Diploids: Salt-stressed medium
(E) Tetraploids: Unstressed medium
(F) Tetraploids: Salt-stressed medium
Genome Size (FL1 Intensity)
Time (Generations)
0
100
200
300
400
600
