# Supplementary material for: Genomic Convergence toward Diploidy in Saccharomyces cerevisiae
Source: PLoS Genet. 2006 Sep 22;2(9):e145. doi: 10.1371/journal.pgen.0020145 (PMC1570378; doi:10.1371/journal.pgen.0020145)
Supplement: Supplementary file 3 [file pgen.0020145.sg003.pdf]

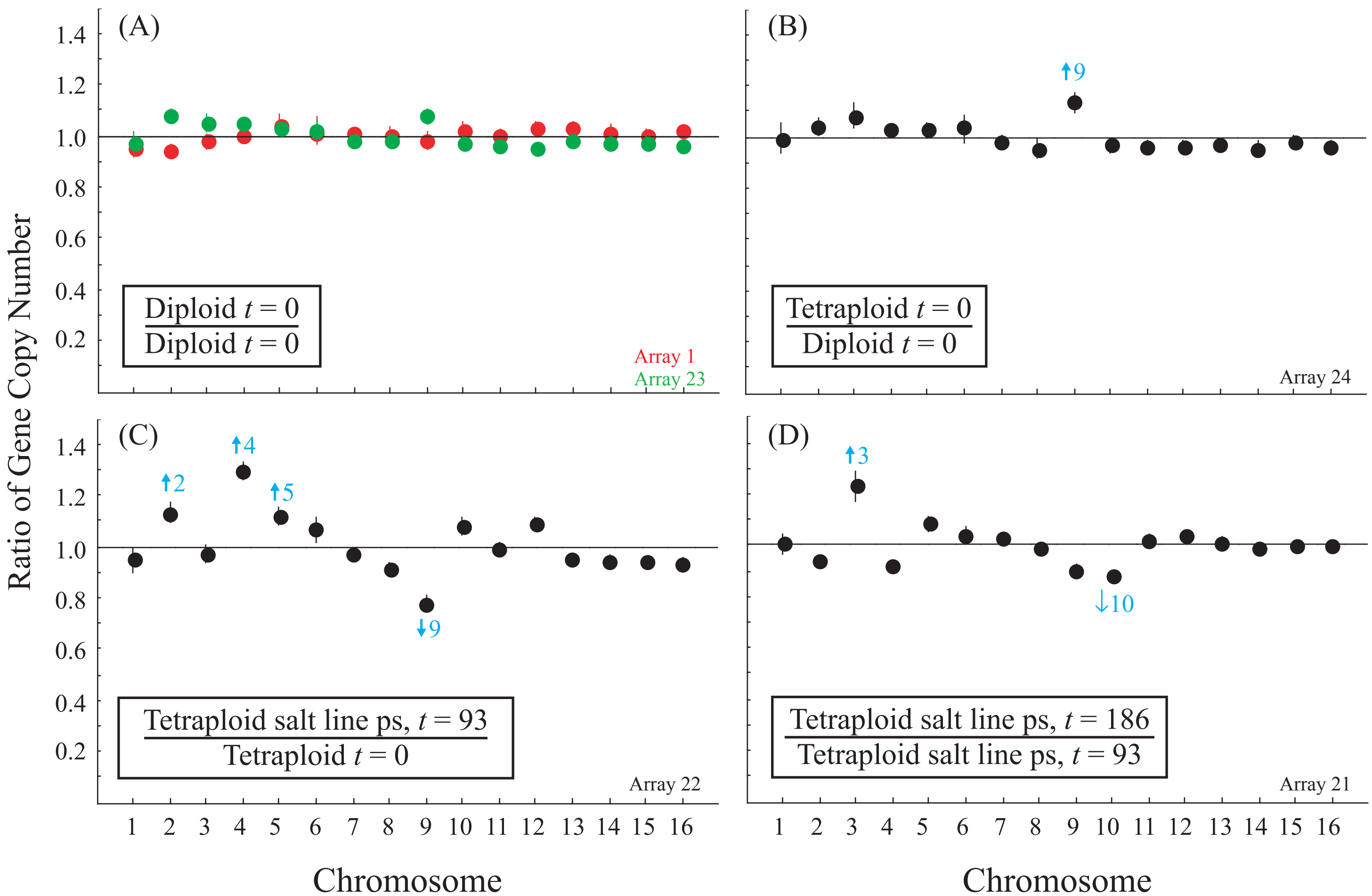

Ratio of Gene Copy Number
(A)
Diploid t = 0 / Diploid t = 0
Array 1
Array 23
(B)
↑9
Tetraploid t = 0 / Diploid t = 0
Array 24
(C)
↑2
↑4
↑5
↓9
Tetraploid salt line ps, t = 93 / Tetraploid t = 0
Array 22
(D)
↑3
↓10
Tetraploid salt line ps, t = 186 / Tetraploid salt line ps, t = 93
Array 21
Chromosome
Chromosome
