# Supplementary material for: Genomic Convergence toward Diploidy in Saccharomyces cerevisiae
Source: PLoS Genet. 2006 Sep 22;2(9):e145. doi: 10.1371/journal.pgen.0020145 (PMC1570378; doi:10.1371/journal.pgen.0020145)
Supplement: Supplementary file 4 [file pgen.0020145.sg004.pdf]

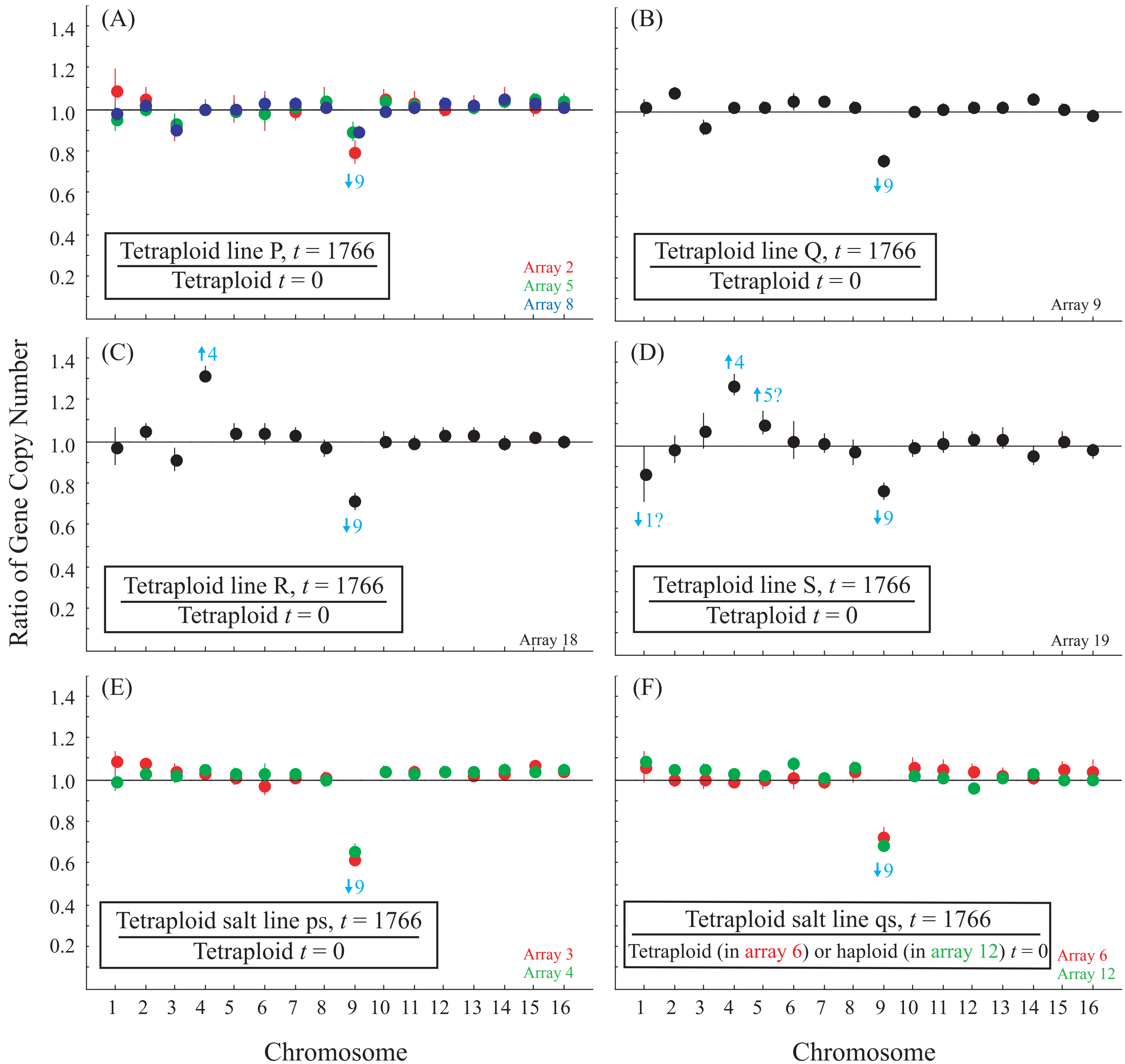

Ratio of Gene Copy Number
(A)
Tetraploid line P, t = 1766
Tetraploid t = 0
Array 2
Array 5
Array 8
(B)
Tetraploid line Q, t = 1766
Tetraploid t = 0
Array 9
(C)
Tetraploid line R, t = 1766
Tetraploid t = 0
Array 18
(D)
Tetraploid line S, t = 1766
Tetraploid t = 0
Array 19
(E)
Tetraploid salt line ps, t = 1766
Tetraploid t = 0
Array 3
Array 4
(F)
Tetraploid salt line qs, t = 1766
Tetraploid (in array 6) or haploid (in array 12) t = 0
Array 6
Array 12
Chromosome
Chromosome
