# Supplementary material for: Genomic Convergence toward Diploidy in Saccharomyces cerevisiae
Source: PLoS Genet. 2006 Sep 22;2(9):e145. doi: 10.1371/journal.pgen.0020145 (PMC1570378; doi:10.1371/journal.pgen.0020145)
Supplement: Supplementary file 5 [file pgen.0020145.sg005.pdf]

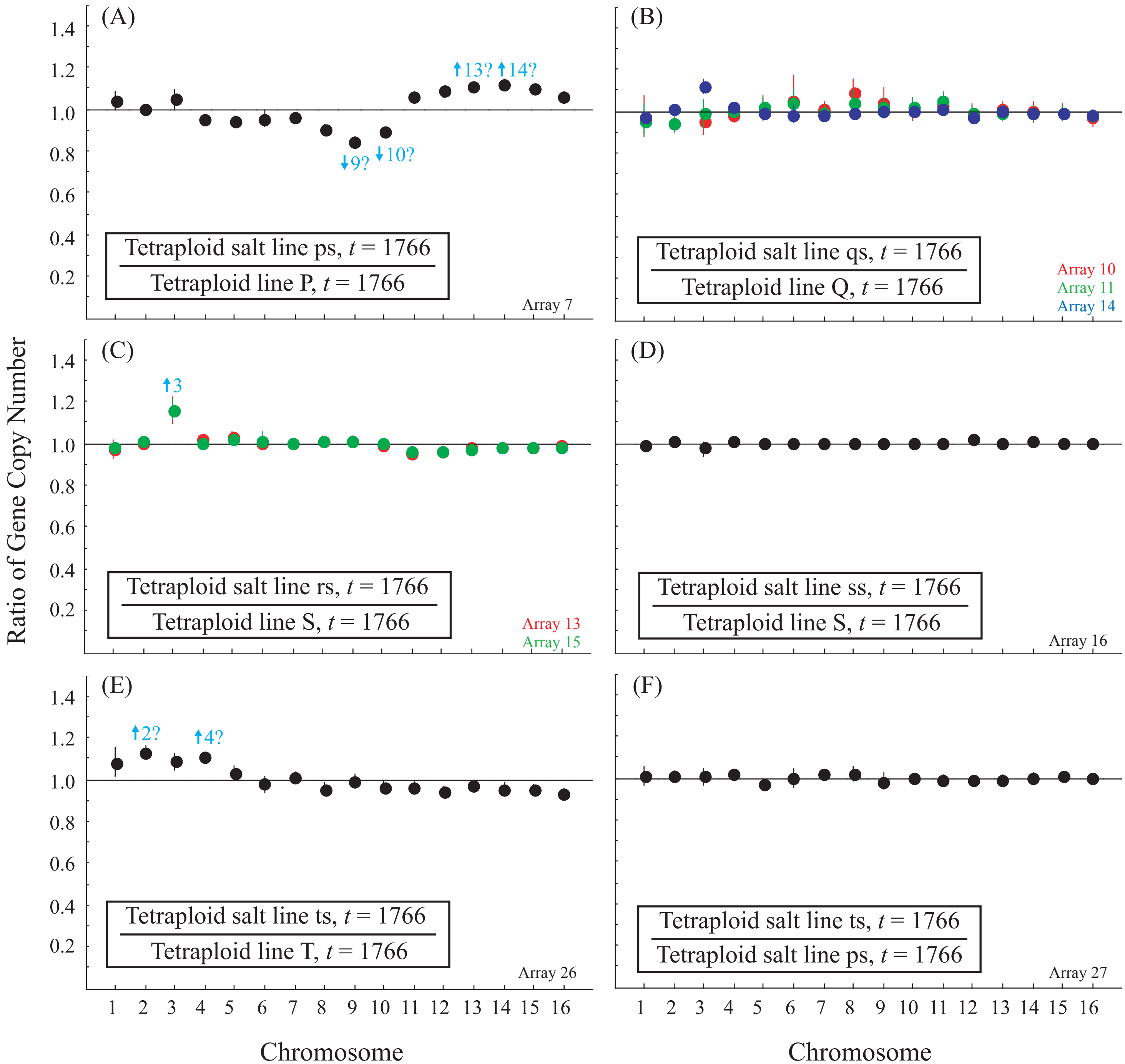

(A)
↑13? ↑14?
↓9? ↓10?
Tetraploid salt line ps, t = 1766
Tetraploid line P, t = 1766
Array 7
(B)
Tetraploid salt line qs, t = 1766
Tetraploid line Q, t = 1766
Array 10
Array 11
Array 14
(C)
↑3
Tetraploid salt line rs, t = 1766
Tetraploid line S, t = 1766
Array 13
Array 15
(D)
Tetraploid salt line ss, t = 1766
Tetraploid line S, t = 1766
Array 16
(E)
↑2? ↑4?
Tetraploid salt line ts, t = 1766
Tetraploid line T, t = 1766
Array 26
(F)
Tetraploid salt line ts, t = 1766
Tetraploid salt line ps, t = 1766
Array 27
Ratio of Gene Copy Number
1.4
1.2
1.0
0.8
0.6
0.4
0.2
1 2 3 4 5 6 7 8 9 10 11 12 13 14 15 16
Chromosome
Chromosome
