# Supplementary material for: Genomic Convergence toward Diploidy in Saccharomyces cerevisiae
Source: PLoS Genet. 2006 Sep 22;2(9):e145. doi: 10.1371/journal.pgen.0020145 (PMC1570378; doi:10.1371/journal.pgen.0020145)
Supplement: Supplementary file 6 [file pgen.0020145.sg006.pdf]

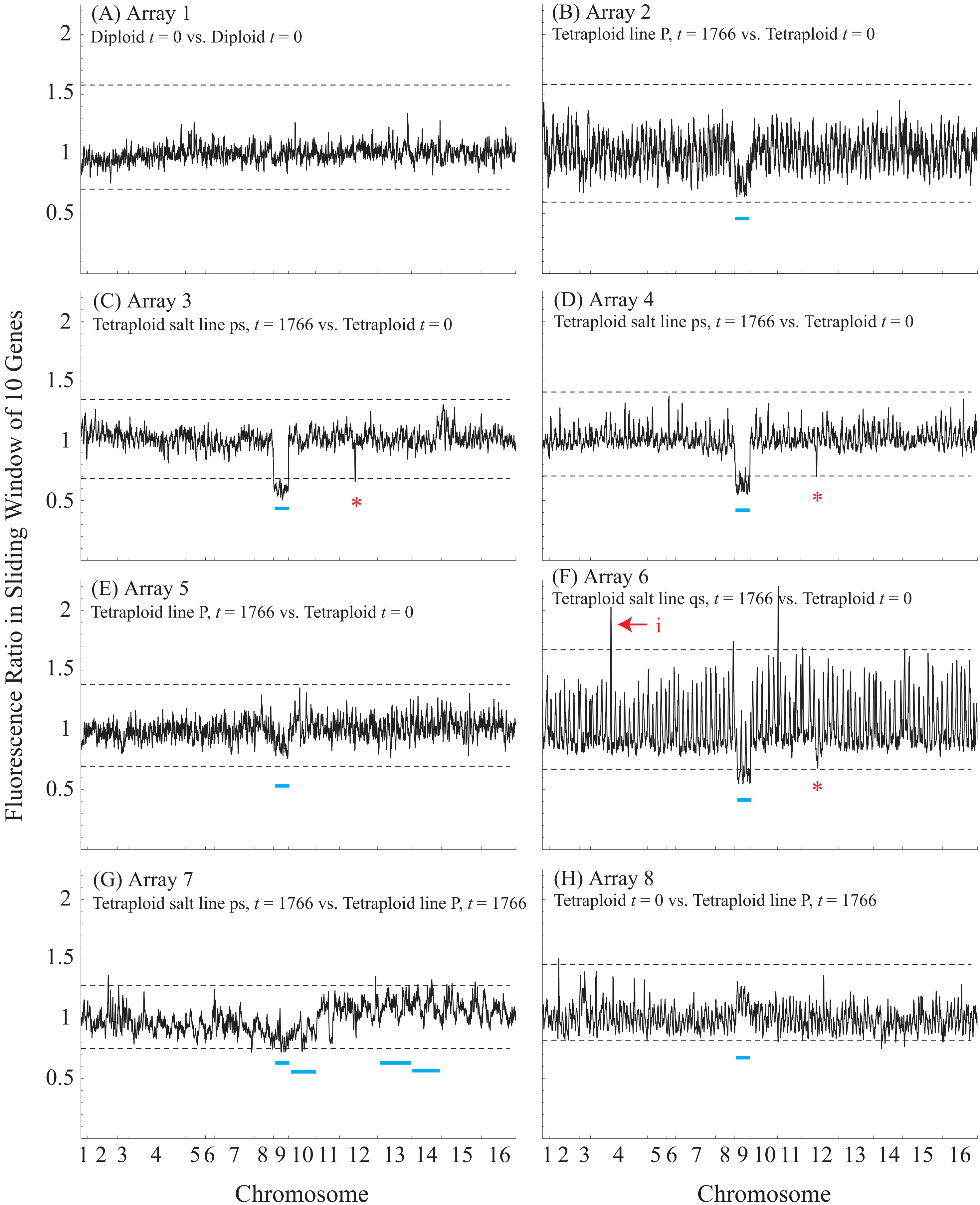

Fluorescence Ratio in Sliding Window of 10 Genes
(A) Array 1
Diploid t = 0 vs. Diploid t = 0
(B) Array 2
Tetraploid line P, t = 1766 vs. Tetraploid t = 0
(C) Array 3
Tetraploid salt line ps, t = 1766 vs. Tetraploid t = 0
(D) Array 4
Tetraploid salt line ps, t = 1766 vs. Tetraploid t = 0
(E) Array 5
Tetraploid line P, t = 1766 vs. Tetraploid t = 0
(F) Array 6
Tetraploid salt line qs, t = 1766 vs. Tetraploid t = 0
i
(G) Array 7
Tetraploid salt line ps, t = 1766 vs. Tetraploid line P, t = 1766
(H) Array 8
Tetraploid t = 0 vs. Tetraploid line P, t = 1766
2
1.5
1
0.5
1 2 3 4 5 6 7 8 9 10 11 12 13 14 15 16
Chromosome

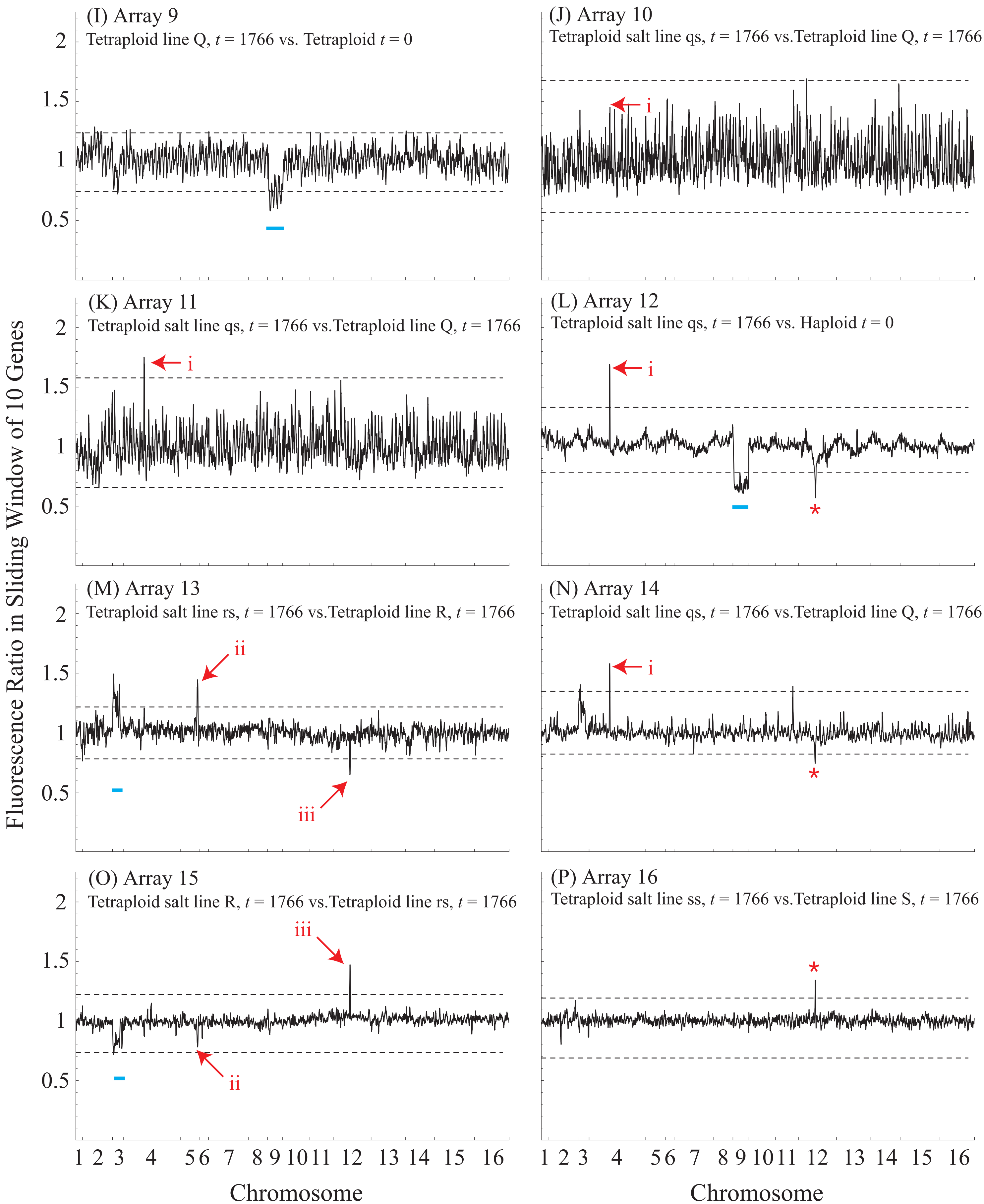
(I) Array 9
Tetraploid line Q, t = 1766 vs. Tetraploid t = 0
(J) Array 10
Tetraploid salt line qs, t = 1766 vs.Tetraploid line Q, t = 1766
(K) Array 11
Tetraploid salt line qs, t = 1766 vs.Tetraploid line Q, t = 1766
(L) Array 12
Tetraploid salt line qs, t = 1766 vs. Haploid t = 0
(M) Array 13
Tetraploid salt line rs, t = 1766 vs.Tetraploid line R, t = 1766
(N) Array 14
Tetraploid salt line qs, t = 1766 vs.Tetraploid line Q, t = 1766
(O) Array 15
Tetraploid salt line R, t = 1766 vs.Tetraploid line rs, t = 1766
(P) Array 16
Tetraploid salt line ss, t = 1766 vs.Tetraploid line S, t = 1766
Fluorescence Ratio in Sliding Window of 10 Genes
Chromosome
i
ii
iii
*
2
1.5
1
0.5
1 2 3 4 5 6 7 8 9 10 11 12 13 14 15 16

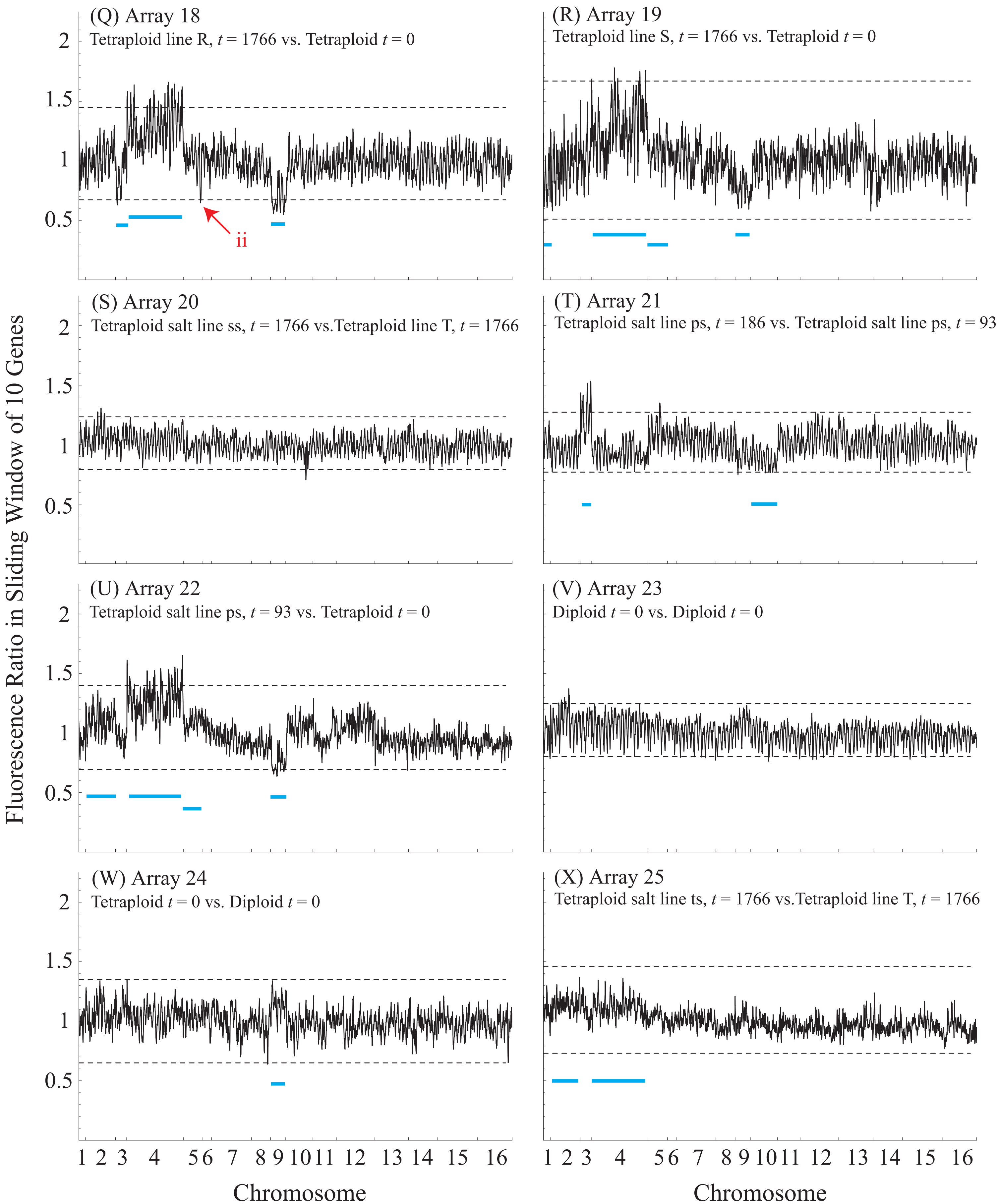

(Q) Array 18
Tetraploid line R, t = 1766 vs. Tetraploid t = 0
ii
(R) Array 19
Tetraploid line S, t = 1766 vs. Tetraploid t = 0
(S) Array 20
Tetraploid salt line ss, t = 1766 vs.Tetraploid line T, t = 1766
(T) Array 21
Tetraploid salt line ps, t = 186 vs. Tetraploid salt line ps, t = 93
(U) Array 22
Tetraploid salt line ps, t = 93 vs. Tetraploid t = 0
(V) Array 23
Diploid t = 0 vs. Diploid t = 0
(W) Array 24
Tetraploid t = 0 vs. Diploid t = 0
(X) Array 25
Tetraploid salt line ts, t = 1766 vs.Tetraploid line T, t = 1766
Fluorescence Ratio in Sliding Window of 10 Genes
2
1.5
1
0.5
1 2 3 4 5 6 7 8 9 10 11 12 13 14 15 16
Chromosome

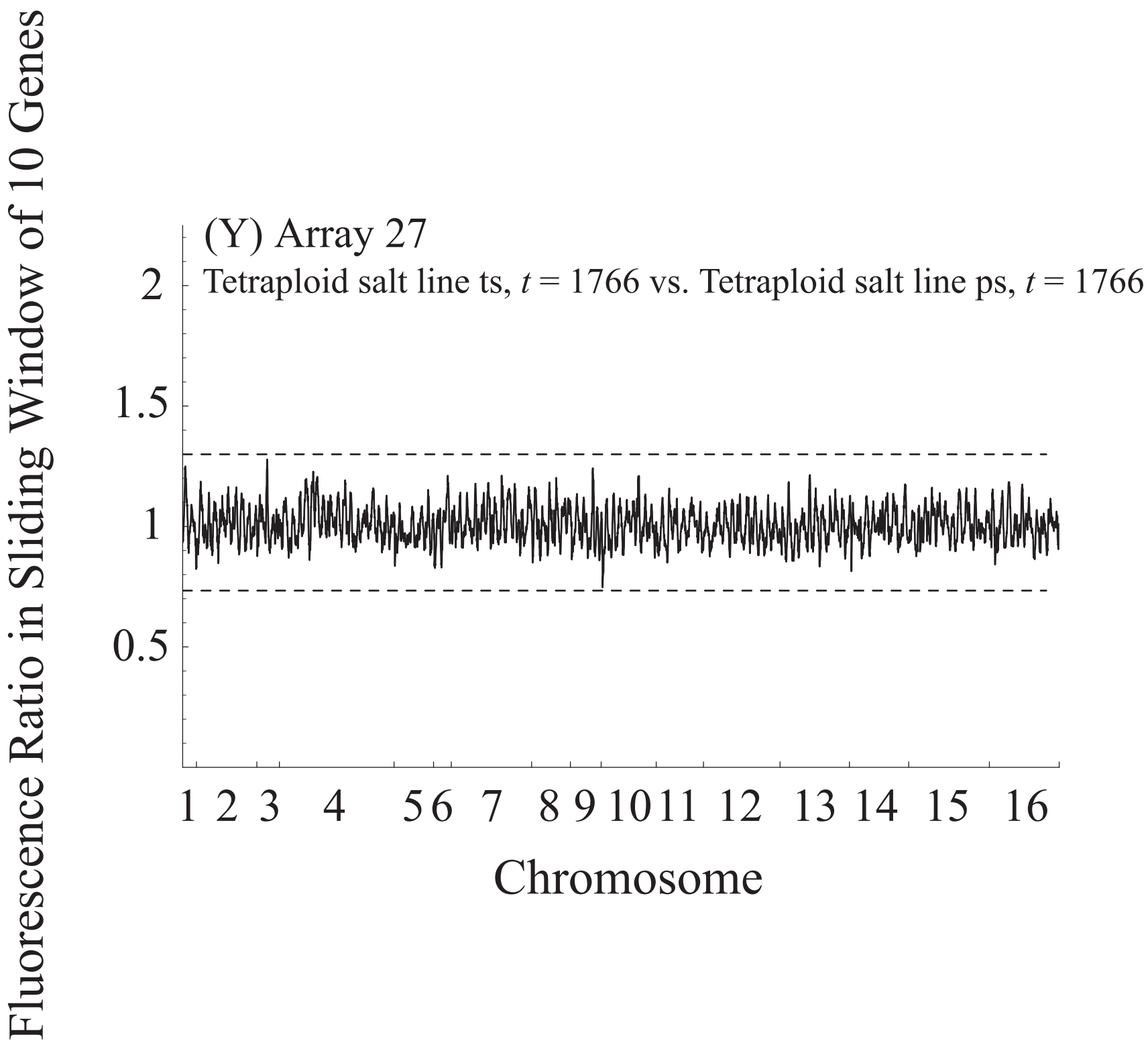
(Y) Array 27
Tetraploid salt line ts, t = 1766 vs. Tetraploid salt line ps, t = 1766
Fluorescence Ratio in Sliding Window of 10 Genes
2
1.5
1
0.5
1 2 3 4 5 6 7 8 9 10 11 12 13 14 15 16
Chromosome
