# Supplementary material for: Genomic Convergence toward Diploidy in Saccharomyces cerevisiae
Source: PLoS Genet. 2006 Sep 22;2(9):e145. doi: 10.1371/journal.pgen.0020145 (PMC1570378; doi:10.1371/journal.pgen.0020145)
Supplement: Supplementary file 7 [file pgen.0020145.sg007.pdf]

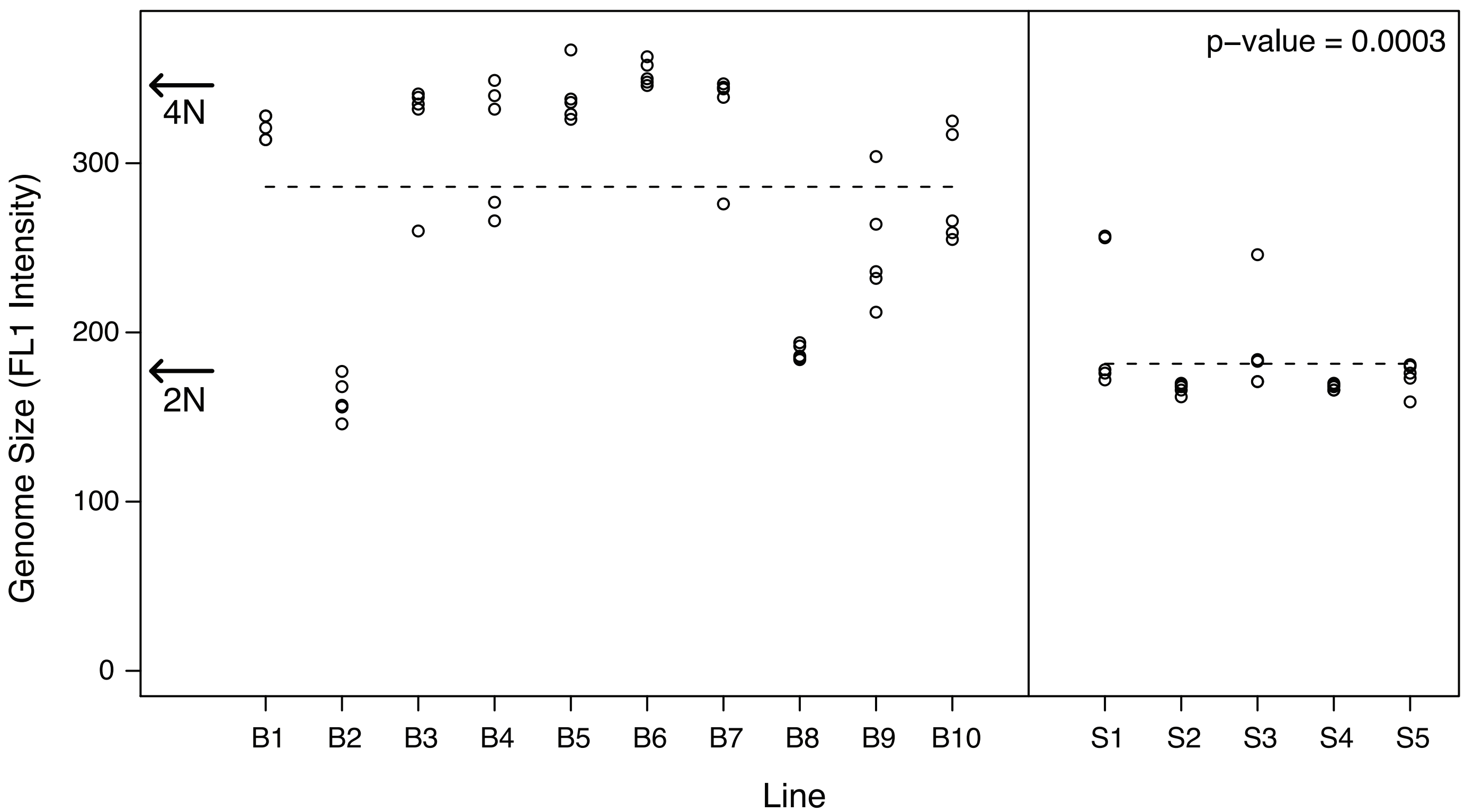

p−value = 0.0003
4N
2N
Genome Size (FL1 Intensity)
0
100
200
300
B1
B2
B3
B4
B5
B6
B7
B8
B9
B10
S1
S2
S3
S4
S5
Line
