# Supplementary material for: Genomic Convergence toward Diploidy in Saccharomyces cerevisiae
Source: PLoS Genet. 2006 Sep 22;2(9):e145. doi: 10.1371/journal.pgen.0020145 (PMC1570378; doi:10.1371/journal.pgen.0020145)
Supplement: Supplementary file 8 [file pgen.0020145.sd001.doc]

Protocol S1

# Replicate Evolution Experiment

30 lines were initiated from the same ancestral stock (BM1N, BM2N, BM4N) and were evolved for 582 asexual generations using the same batch culture evolution methodology as the primary experiment. Results from FACScans are shown in Figure S1.

# Analysis of Aneuploidy by CGH

Comparative genomic hybridization (CGH) was performed using 27 microarrays. Some arrays that appeared nosiy after intial analysis (e.g. array 10) were repeated. Otherwise, we attempted to maximize the number of lines hybridized onto the arrays using a round robin appraoch. All 25 successful arrays are shown (hybridization failed on arrays 17 and 25). Supplementary Figures S3 – S5 show the average fluorescence ratio of each chromosome obtained by CGH, along with 99.8% confidence intervals obtained by bootstrapping all of the gene ratio data from the particular chromosome.

Figure S3 compares genomes early in the experiment. Figure S3a compares two diploid lines from time point 0. The small amount of variation from the expected ratio of one is unlikely to be biologically significant. For example, consider the puzzling observation that numerically similar chromosomes (e.g., chromosomes 10 – 16) differ significantly from the expected ratio of one in the same direction (see green array 23 in Figure S3a). This observation is likely explained by the fact that genes on the same chromosome and from numerically adjacent chromosomes are more likely to be within the same row or column on the microarrays provided by the University Health Network. This interpretation is supported by the fact that the confidence intervals approximately double in length if we bootstrap by block (i.e., sample with replacement among the 48 blocks of 16$´$17 spots), a procedure that controls for the non-random design of the array within blocks. As aneuploidy should cause downward shifts to 0.75 (loss of one chromsomome in a tetraploid) or upward shifts to 1.25 (gain of one chromosome in a tetraploid), we ignore small deviations from one (0.9 – 1.1) and discuss only larger departures.

Using this cut-off, chromosome 9 is at an average ratio of 1.14 in a tetraploid-diploid comparison at time 0 (Figure S3b). Even more variation is observed between the ps tetraploid line at cell generation ~100 and at generation 0 (Figure S3c), with chromosomes 2, 4, 5, and 9 at ratios 1.13, 1.29, 1.12, and 0.77, respectively. Similarly, substantial variation is observed between a ps tetraploid line at generation 200 and at generation 100, with chromosomes 3 and 10 at ratios 1.23 and 0.88, respectively. Most of these differences fall short of the expected ratios of 0.75 and 1.25 for tetraploid lines that gain or lose a chromosome. Two biologically interesting explanations are possible: partial aneuploidy or polymorphism within the lines compared by CGH. Sliding window analyses of these chromosomes showed no evidence of partial chromosome loss. Another possibility is that the lines had become polymorphic for chromosome content during the approximately 30 cell generations required to isolate significant DNA from cultures starting from a single cell.

Figure S4 illustrates CGH comparisons between lines isolated at generation 1766 and at generation 0. A majority of chromosomes exhibit fluorescence ratios near one, with some exceptions. Interestingly, aneuploidy always involved chromosome 9 (ratios of 0.86, 0.76, 0.71, 0.78, 0.64, and 0.70 from a - f), and often involved chromosome 4 (ratios of 1.32 and 1.28 from middle left to middle right).

Figure S5 illustrates CGH comparisons between different tetraploid lines isolated at generation 1766. Variation in chromosomal content is much less pronounced among comparisons made at the end of the evolution experiment than between lines at the start. In array 7, chromosomes 3, 9, 13 and 14 showed deviations beyond 0.9 – 1.1 (Figure S5a), as did chromosomes 2 and 4 in array 26 (Figure S5e). Yet these arrays also exhibited the pattern that numerically similar chromosomes differed in the same direction, suggesting that these deviations were artifacts. Indeed, sliding window analyses (Figure S6) did not show clear breaks at the chromosome boundaries, as expected from a chromosomal addition/deletion. The only consistent difference in genomic content among the lines at the end of the experiment was chromosome 3 in the comparisons between line rs19 and R19 in arrays 13 and 15 (Figure S5c; average ratio = 1.15). Sliding window analyses of arrays 13, 15, and 18 suggest a partial deletion of chromosome 3 in line R19.

The interpretation of the microarrays is made complicated by the fact that the time 0 “controls” exhibited variation in chromosomal content (Figure S3). Nevertheless, several broad patterns are apparent in the results. Most importantly, the roughly triploid or diploid DNA content of the evolved lines detected by FACS analysis (Figure 1) is not consistent with random chromosome loss, as most of the lines analyzed from generation 1766 exhibited chromosomal ratios near one. However, the lines do exhibit aneuploid differences, appearing early in the experiment and often involving chromosome 9.

# Analysis of Indels by CGH

CGH was also used to detect smaller deletions and insertions. We performed sliding window analyses using a window size of 10 genes to search for indels. We only focused on those windows that showed peaks or troughs that were outside of the range observed in 100 randomizations of the genome and that were consistent across multiple arrays involving the same lines. Using these criteria, three potential indels were identified. Multiple arrays allowed us to pinpoint the type of indel that occurred in case (i); our inferences for cases (ii) and (iii) are based on fewer arrays and are more tentative.

(i) Chromosome 4 insertion: A sliding window analysis of arrays 6 and 12 (designated *i* in Figure S6) demonstrated a higher ratio of the same segment on chromosome 4 (525434..538461) in qs-1766 relative to Q-1766. The genes involved, *KRS1*, *ENA1, ENA2,* and *ENA5*, were found at ratios in this region of 3.7 [2.5,5.6] and 3.1 [2.0-4.7] with 95% confidence intervals in brackets (based on a normal approximation using observed standard errors for the log-ratio data). Arrays 10, 11, and 14 also exhibit higher ratios in this region at 2.8 [1.2-6.4] (array 10), 4.0 [2.0-8.1] (array 11), and 2.6 [2.0-3.4] (array 14), indicating that qs-1766 carries an insertion. Consistent with this conclusion, the ratio in this region was not significantly different from one in array 9 (0.94 [0.73-1.13]) involving line Q-1766. We thus infer that an insertion has occurred in the qs high-salt line, increasing the copy number of these genes several fold. Interestingly, *ENA5*, *ENA2*, and *ENA1* are P-type ATPases involved in Na+ and Li+ efflux (*Saccharomyces* Genome Database: http://www.yeastgenome.org/). Transposable element (TE) disruption within *ENA5* and *ENA1* has been shown to decrease salt tolerance[1]. We hypothesize that duplication of this region was selectively favoured in the high salt medium, improving the salt tolerance of the qs line.

(ii) Chromosome 5 indel: A sliding window analysis of array 13 and, to a lesser extent, array 15 (designated *ii* in Figure S6) indicated a lower ratio of a segment on chromosome 5 (spanning approximately sites 451560..487188 and encompassing ~18 genes) in R-1766 relative to rs-1766 (ratio 0.721 [0.68-0.74] in array 13; ratio 0.81 [0.78-0.83] in array 15). A similar trend was observed in array 18 (ratio of 0.68 [0.64-0.73] for R-1766 relative to a tetraploid line from time point 0), suggesting a deletion in R-1766, but the array was noisier (Figure S6q). Several genes within this region are known to reduce growth on YPD when deleted, either as heterozygous deletions *COG3* or as homozygous deletions (*COX15*, *OXA1*, *PET122*, *UBP3*, *BEM2*, *COG3*)[2], which argues against selective benefit to this deletion. Confirmation of the deletion and quantification of its fitness effects are warranted.

(iii) Chromosome 12 indel: A sliding window analysis of arrays 13 and 15 (designated *iii* in Figure S5) demonstrated a significantly lower ratio of a segment on chromosome 12 (469318..489930) in rs-1766 relative to R-1766. The genes involved, *ASP3-1*, *RDN5-3*, *ASP3-2*, *RDN5-4*, *RDN5-5, ASP3-4*, *RDN5-6*, were found at ratios of 0.60 [0.55-0.67] (array 13) and 0.64 [0.60-0.69] (array 15). No such pattern was observed in a sliding window analysis of R-1766 versus a time point 0 strain (1.3 [1.0-1.6], array 18), suggesting a deletion of this segment in rs-1766. These genes either have unknown function or are cell-wall proteins involved in asparagine catabolism (*Saccharomyces* Genome Database: http://www.yeastgenome.org/).

Interestingly, sliding window analyses suggest the presence of indels involving the same region of chromosome 12 in other genomic comparisons (asterisks in Figure S6), including line ps-1766 vs ancestral (arrays 3 and 4), ss-1766 vs S-1766 (array 16), qs-1766 vs time 0 or Q-1766 (arrays 6, 12, 14). These signals were not, however, detectable across all arrays involving the same lines. This inconsistency might be due to the difficulties of detecting small indels using CGH and/or due to polymorphisms arising repeatedly in the preparation of DNA if this is an indel hotspot.

**Bottleneck Experiment**

Genome size was measured for ten initially tetraploid populations (B1-B10) evolved by repeated bottlenecking from colonies down to single cells for 566 cell generations. The genome size of five individuals were sampled from each line and compared to five individuals from each of the tetraploid populations evolved through batch culture transfers for 558 generations (from the orignial experiment). The population averages were significantly higher (p=0.0003; Figure S7) in the bottleneck line.

**Calculating required fitness gains**

We observed transitions from tetraploid (4n=64 chromosomes) to diploid (2n = 32 chromosomes) in replicate populations over the course of 100 – 200 generations (Figure 1e). We hypothesize that these transitions were driven by large-scale reductions in genome size, involving more than one chromosome at a time. Here, we show that it would take extremely strong selection if the transition instead involved a series of 32 mutations, each involving the loss of a single chromosome.

In the absence of sex and recombination, successive beneficial mutation can only spread to fixation if they arise sequentially in the same background. Following Crow & Kimura[3], we calculate the expected number of generations between the appearance of the first beneficial mutation in an asexual population and the appearance of an individual that carries two beneficial mutations, counting only those beneficial mutations that survive stochastic loss while rare. At generation *t*, we expect *N p(t)* μ second mutations to occur in an individual carrying the first mutation, where *p(t)* is the frequency of the first mutation, *N* is the population size, and μ is the rate at which the second mutation occurs. Of these second mutations, only a fraction will survive loss while rare[4]; when selection is strong, the fraction surviving is approximately $1-e^{-2s}$.

We can then ask how many generations, *g*, must pass before the cumulative expected number of second mutations (specifically, beneficial mutations that arise in an individual carrying the first mutation and surviving loss while rare) rises above one. That is, we find *g* such that $\int Np\left( t \right)m\left( 1-e^{-2s} \right)dt>1$. Measuring time from the appearance of the first mutation, the frequency of the first mutation obeys the classical model of selection: $p\left( t \right)=\left( 1+s \right)^{t}p\left( 0 \right)/\left( 1-p\left( 0 \right)+\left( 1+s \right)^{t}p\left( 0 \right) \right)$, where $p\left( 0 \right)=1/N$. Solving the integral, we find:

$g=\frac{\text{ln}\left( N\left( 1+s \right)^{1/\left( Nm\left( 1-e^{-2s} \right) \right)}-N+1 \right)}{\text{ln}\left( 1+s \right)}$	(S1)

Equation (S1) gives the expected number of generations until two beneficial mutations are present together in the same individual. Consequently, 31$´$*g* generations must pass, on average, until an individual would arise that had lost 32 chromosomes by mutation, if each mutation were to occur independently. In our experiment, the effective population size was on the order of *N* = 10^7^. Using this parameter value and a relatively high mutation rate of μ =10^-5^ , we find that the selection coefficient, *s*, must be greater than 5.5 to account for a transition from tetraploidy to diploidy in 200 generations by the loss of one chromosome at a time. This corresponds to a 550% increase in fitness every time a chromosome is lost. This estimated selection coefficient is not sensitive to the exact value of the population size, and much higher mutation rates are needed to cause the requisite selection coefficient to drop substantially (e.g., μ >10^-3^ for *s* < 2). Although we have treated the population size as constant, accounting for fluctuations in population size only serves to increase the selection coefficient needed to account for 32 independent mutations arising and spreading in succession (details available upon request). It is highly implausible that the loss of each chromosome is accompanied by such extreme selection, leading us to conclude that the transition from tetraploidy to diploidy did not occur by a series of independent mutations, losing one chromosome at a time. Instead, our data support the hypothesis that multiple chromosomes were lost simultaneously. Whether or not this was precipitated by a mutational event (e.g., losing one chromosome increases the mutation rate to loss of non-homologous chromosomes) remains unknown.

**Supplementary Information References:**

1. de Jesus Ferreira, M.C., Bao, X., Laizé, V., & Hohmann, S. Transposon

mutagenesis reveals novel loci affecting tolerance to salt stress and growth at low temperature. *Curr Genet.* **40**:27-39 (2001)

2. Deutschbauer A. M *et al.* Mechanisms of haploinsufficiency revealed by genome-

wide profiling in yeast. *Genetics* **169**:1915-25 (2005)

3. Crow, J.F. & Kimura, M. Evolution in sexual and asexual populations. *Am Nat* **99**:

439-450 (1965)

4. Haldane, J.B.S. The mathematical theory of natural and artificial selection. *Proc.*

*Camb. Philos. Soc.* **23**: 838-844 (1927)

**Supplementary Figure S1**: Temporal polymorphism for genome size across 1800 generations of batch culture. One line from each hapoid and tetraploid population was picked for more in depth analysis. 10 colonies at each time point were assayed for genome size (closed circles). The line and open circles denote the original, single colony data presented in Figure 1.

**Supplmentary** **Figure S2:** Genome size change across ~600 generations of batch culture evolution from a replicate experiment. FL1 is a linear scale of dye fluorescence as measured by flow cytometry (FACS). The five lines on each graph represent the five replicate lines evolved independently. Note the similar pattern of genome size change depicted in this figure and the first ~600 generations of Figure 1.

**Supplementary Figure S3:** Average fluorescence ratio by chromosome from a comparative genomic hybridization between lineages early in the experiment. Repeated arrays are illustrated in different colors. Chromosomes with average fluorescence ratios outside of 0.9 – 1.1 are indicated in blue text.

**Supplementary Figure S4:** Average fluorescence ratio by chromosome from a comparative genomic hybridization between lineages late and early in the experiment. Repeated arrays are illustrated in different colors. Chromosomes with average fluorescence ratios outside of 0.9 – 1.1 are indicated in blue text.

**Supplementary** **Figure S6:** Fluorescence ratios for sliding window analyses of the arrays, using a window length of 10 genes. Dashed lines represent the maximum and minimum observed in sliding windows of 100 randomized genomes. Blue lines represent chromosomal differences inferred from Figures S1 – S3. Letters i - iii (and *) represent indels discussed in the text*.*

**Supplementary** **Figure S7:** Genome size measured from ten populations (B1-B10) evolved by repeated bottlenecking for 566 generations and five populations (S1-S5) evolved through batch culture transfers for 558 generations. Five individuals were sampled from each population (circles). The population averages were significantly higher (p=0.0003) for the bottleneck lines (B1-B10) than the original batch culture lines (S1-S5). The two arrows pointing at the y-axis indicate the average genome size (±2.5) of five individuals sampled from the ancestral diploid and tetraploid stocks.
